# Association Between Systemic Arterial Stiffness and Choroidal Vascular Resistance Assessed by Laser Speckle Flowgraphy in Diabetic Retinopathy

**DOI:** 10.1167/tvst.15.10.41

**Published:** 2026-10-26

**Authors:** Naoki Fujioka, Kazufumi Tanaka, Moe Nunose, Sara Imai, Ryota Takenaka, Fumihiko Yagi, Yuichi Hori, Ryuya Hashimoto

**Affiliations:** 1 Department of Ophthalmology, Toho University Graduate School of Medicine, Tokyo, Japan; 2 Department of Ophthalmology, Toho University Sakura Medical Center, Chiba, Japan

**Keywords:** diabetic retinopathy, Choroid, blood flow, cardio ankle vascular index, laser speckle flowgraphy

## Abstract

**Purpose:**

This study aimed to investigate the association between peripapillary choroidal hemodynamics, as measured by laser speckle flowgraphy (LSFG), and systemic arterial stiffness, indicated by the cardio-ankle vascular index (CAVI), in patients with diabetic retinopathy (DR).

**Methods:**

In this retrospective cross-sectional study, 72 eyes from 72 patients with type 2 diabetes across DR stages were analyzed. Choroidal parameters, including mean blur rate (MBR; indicating blood flow), beat strength (BS; reflecting pulsatility), and the BS/MBR ratio (BOM; a resistance-related waveform index), were quantified in both temporal and nasal peripapillary regions. A stepwise multiple regression analysis was conducted to identify independent predictors of CAVI, considering age, sex, and LSFG parameters.

**Results:**

In univariate analyses, higher CAVI was significantly associated with increased choroidal pulsatility and resistance (*P* < 0.05). In the final exploratory stepwise multivariable model, age remained significantly associated with higher CAVI (β = 0.048, *P* = 0.020), while temporal BOM showed a borderline positive trend with CAVI (β = 2.719, *P* = 0.055). The model was statistically significant overall (adjusted *R*² = 0.326, *P* < 0.001).

**Conclusions:**

In patients with DR, age was the most robust determinant of CAVI. The retention of regional choroidal BOM parameters in the final exploratory stepwise model suggests a possible association whereby systemic pulsatile stress may influence choroidal hemodynamics, although these findings are exploratory and hypothesis-generating.

**Translational Relevance:**

LSFG-derived choroidal resistance (BOM) may help explore the ocular–systemic vascular connection in diabetes management.

## Introduction

Diabetic retinopathy (DR) is a significant microvascular complication of diabetes mellitus and a leading cause of vision impairment globally.^1^ Although it is fundamentally a vascular disease, its pathogenesis extends beyond the well-documented changes in retinal vasculature. Recent evidence increasingly emphasizes the critical role of alterations in choroidal circulation, referred to as diabetic choroidopathy, in the progression of the disease.^2^ Histological and angiographic studies have identified multiple choroidal abnormalities in diabetic eyes, including narrowing and dropout of the choriocapillaris, vascular remodeling, and regions of non-perfusion.^2^ Furthermore, functional studies using color-flow Doppler imaging have revealed impaired choroidal circulation, evidenced by an increased resistivity index, even in patients without clinically apparent DR.^3^ These findings suggest that hemodynamic compromise in the choroid may precede or exacerbate retinal pathology.

Moreover, DR is recognized not merely as an isolated ocular condition but as a manifestation of systemic vascular dysfunction, particularly characterized by increased arterial stiffness.^4^ The choroid, being a highly vascularized and high-flow structure, is less influenced by local metabolic signals compared to the retinal circulation. Instead, it relies significantly on neural control and perfusion-pressure regulation, rendering it potentially susceptible to systemic hemodynamic stress.^5^ The cardio-ankle vascular index (CAVI) has emerged as a robust, noninvasive measure of systemic arterial stiffness from the aorta to the ankle.^6^ A notable advantage of CAVI is its theoretical independence from blood pressure at the time of measurement, providing a more stable assessment of the intrinsic biomechanical properties of the arterial wall.^6^^,^^7^ Although elevated CAVI values are predictive of future cardiovascular events^8^ and correlate with coronary atherosclerosis,^9^ their impact on the delicate ocular microvasculature remains poorly understood. In patients with diabetes, higher CAVI values have been associated with increased DR severity and structural changes, such as a reduced choroidal vascularity index,^10^^,^^11^ suggesting a mechanical link between macrovascular stiffening and microvascular damage.

Laser speckle flowgraphy (LSFG) is a noninvasive technology that facilitates real-time, quantitative assessment of microcirculation in the ocular fundus, including the choroid.^12^^–^^19^ This technique generates several hemodynamic parameters. Although early pulse waveform metrics such as blowout time and skew were used to evaluate pulsatility and vascular resistance, newer analyses have produced more robust indexes. These include beat strength (BS), a pulsatility index derived from frequency analysis that does not require individual heartbeat detection, and beat strength (BS) over mean blur rate (MBR), a resistance-related LSFG waveform index conceptually comparable to Doppler-derived pulsatility or resistive indexes.^20^^,^^21^ Foundational work by Shiba et al. initially established a direct correlation between older LSFG waveform parameters (blowout time and skew) and systemic vascular indices, including CAVI.^22^^,^^23^ This research demonstrated that large arterial function significantly influences ocular microcirculatory hemodynamics.^23^

Despite the established link between systemic stiffness and DR, a comprehensive investigation of the relationship between CAVI and choroidal hemodynamic parameters—particularly the newer BS and BOM indexes—in patients specifically with DR is lacking. Understanding this relationship is crucial for elucidating the hemodynamic mechanisms underlying diabetic choroidopathy. However, the precise impact of macrovascular stiffening on region-specific choroidal resistance remains understudied. Addressing this gap is essential for the development of non-invasive ocular biomarkers for systemic vascular health. Therefore this study aimed to investigate this association, hypothesizing that increased systemic arterial stiffness exerts quantifiable hemodynamic stress on the choroid, manifesting as altered vascular resistance.

## Material and Methods

### Study Design and Participants

This retrospective cross-sectional study was conducted at the Department of Ophthalmology, Toho University Sakura Medical Center. The study protocol received approval from the Institutional Ethics Committee of the Faculty of Medicine, Toho University (approval number: T2024-4180; approval date: November 6, 2025) and conformed to the tenets of the Declaration of Helsinki. Given the study's retrospective design, the requirement for written informed consent was waived, and an opt-out procedure was implemented, allowing potential participants to decline inclusion.

We analyzed data from 72 right eyes of 72 patients (41 men, 31 women; mean age: 61.2 ± 14.3 years) diagnosed with type 2 diabetes mellitus. To avoid intereye correlation, only one eye per participant was included.^24^ The right eye was prespecified as the study eye according to a predefined laterality rule. This approach was also supported by our recent finding that bilateral LSFG-derived choroidal blood flow did not differ significantly between eyes.^25^

Participants were stratified into five groups according to the International Clinical Diabetic Retinopathy Disease Severity Scale: no DR (*n* = 10), mild nonproliferative DR (NPDR, *n* = 9), moderate NPDR (*n* = 9), severe NPDR (*n* = 5), and proliferative DR (PDR, *n* = 39). Inclusion criteria encompassed a confirmed diagnosis of type 2 diabetes, age ≥ 18 years, and the availability of comprehensive ophthalmic and systemic data. Exclusion criteria included atrial fibrillation (which precludes accurate measurement of CAVI), active uveitis, glaucoma, non-diabetic optic neuropathy, high myopia (spherical equivalent < −6.0 diopters), and a history of vitreoretinal surgery or recent (within the past three months) intravitreal injections of anti-vascular endothelial growth factor agents or steroids. Additionally, eyes with significant media opacities that hindered reliable imaging were excluded.

### Ophthalmic and Systemic Examinations

All participants underwent a comprehensive ophthalmic examination, which included the measurement of best-corrected visual acuity using a standard Snellen chart (converted to the logarithm of the minimum angle of resolution [logMAR] for analytical purposes), intraocular pressure measurement via Goldmann applanation tonometry, slit-lamp biomicroscopy, and a dilated fundus examination. The severity of DR was independently graded by three retina specialists (R.H., N.F., and K.T.).

Systemic hemodynamic parameters, including systolic and diastolic blood pressure (SBP, DBP) and heart rate, were recorded following a minimum of 10 minutes of rest in a seated position. Baseline laboratory data, encompassing glycated hemoglobin, lipid profiles, and renal function tests, were retrieved from medical records within one month preceding the ophthalmic examination. Body mass index (BMI) was computed by dividing weight (kg) by height squared (m²).

### Assessment of Systemic Arterial Stiffness

Systemic arterial stiffness was evaluated using the CAVI, measured with a vascular screening system (VaSera VS-3000; Fukuda Denshi, Tokyo, Japan). Measurements were conducted by trained technicians in a temperature-controlled environment after the patient had rested for at least 10 minutes in the supine position. The CAVI serves as an indicator of arterial stiffness from the origin of the aorta to the ankle and is theoretically independent of blood pressure at the time of measurement.^6^^,^^7^ For consistency with the prespecified right-eye analysis, the CAVI value from the right side was used for analysis to enable laterality-matched comparisons between ocular and systemic measurements.

### Data Collection

Baseline data were extracted from electronic medical records and included patient demographics (age, sex), systemic blood pressure (SBP, DBP), and laboratory results from fasting blood samples (hemoglobin A1c, lipid profiles, and renal function tests). BMI was also calculated. In addition, we extracted data regarding routine ophthalmic examinations, including imaging studies such as LSFG, and systemic vascular assessments (CAVI), which were conducted as standard clinical management for diabetic complications. From these clinical records, we retrospectively identified and analyzed patients who had temporally matched LSFG and CAVI data collected within a three-month window to ensure hemodynamic comparability.

### Choroidal Blood Flow Imaging and Analysis

Choroidal hemodynamics were quantitatively assessed using LSFG-NAVI (Softcare Co., Ltd., Fukuoka, Japan). Pupils were dilated with 0.5% tropicamide and 0.5% phenylephrine hydrochloride 20 minutes before imaging.

Quantitative analysis was conducted using LSFG Analyzer software (version 3.14.1.0; Softcare Co., Ltd.). Two distinct peripapillary choroidal regions were analyzed: a temporal region centered on the macula and a nasal peripapillary region (Fig. 1). Because the choroidal circulation is segmentally supplied by the posterior ciliary arteries and contains watershed zones, these temporal and nasal regions were predefined to account for potential topographic differences in peripapillary choroidal hemodynamics.^26^ Our recent study further showed that the spatial pattern of the choroidal watershed zone varies among eyes and that choroidal hemodynamic parameters differ between the CHZ and adjacent choroid, supporting local heterogeneity in peripapillary choroidal circulation.^25^

**Figure 1.**
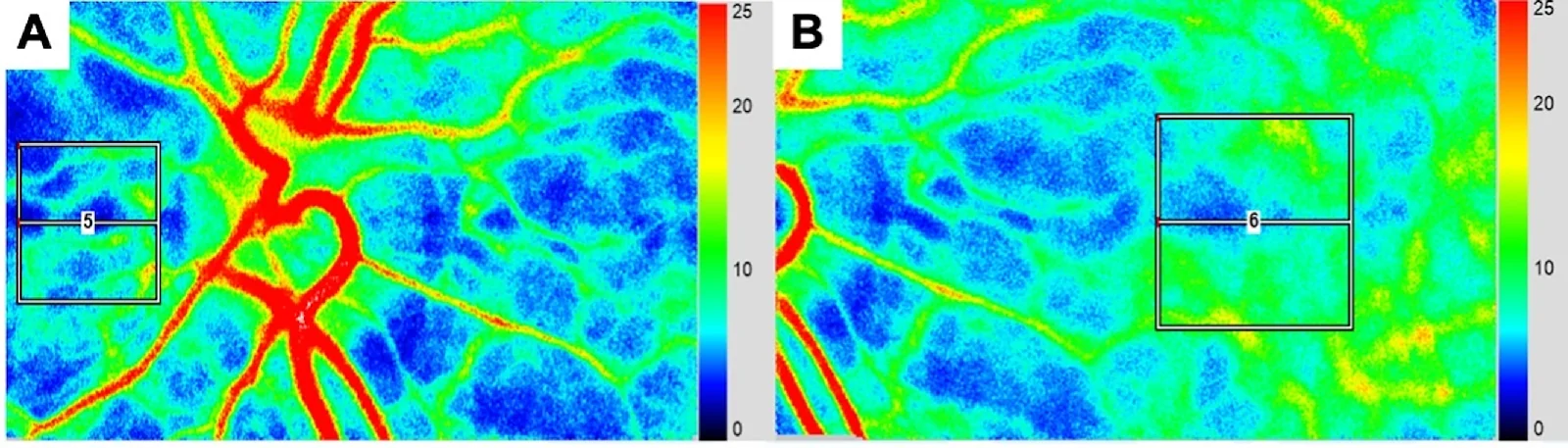
Choroidal blood flow and pulsatility analysis. Representative laser speckle flowgraphy color maps illustrating the placement of ROIs in the peripapillary choroid. (**A**) A square ROI (150 × 150 pixels) is positioned in the temporal peripapillary region, centered on the macula. (**B**) A corresponding ROI is positioned in the nasal peripapillary region. Care was taken to avoid large retinal vessels to isolate choroidal blood flow. The color scale represents the mean blur rate, with *red* indicating a higher blood flow velocity.

A square region of interest (ROI) measuring 150 × 150 pixels was designated at each location, with careful avoidance of large retinal vessels to isolate choroidal flow. ROI placement was performed by a retina specialist and single examiner (N.F.) using this predefined square template to ensure procedural consistency and avoid inter-examiner variability. The following hemodynamic parameters were derived from the heartbeat-synchronized MBR waveform:

• MBR: A quantitative index of relative erythrocyte velocity, representing overall tissue blood flow.^12^
• BS: An index of pulsatility derived from Fourier analysis of the MBR waveform, indicating the amplitude of blood flow fluctuations between systole and diastole.^20^
• BOM: An LSFG-derived resistance-related waveform index calculated as BOM = BS/mean MBR. Because BOM is expressed as a ratio, it may be interpreted as a waveform parameter partially normalized to flow magnitude rather than a direct physical measurement of vascular resistance. This parameter is conceptually analogous to the resistive index or pulsatility index used in Doppler flowmetry.^20^^,^^21^
• Low frequency (LF): A parameter reflecting blood flow fluctuations in the low-frequency band (0.07–0.31 Hz), potentially indicative of autonomic vasomotor regulation.^27^

### Statistical Analysis

Statistical analyses were conducted using R software (Version 4.3.1; R Foundation for Statistical Computing, Vienna, Austria). Continuous variables are expressed as the mean ± standard deviation. To assess differences in continuous variables across the five DR severity groups, one-way analysis of variance was used. When significant differences were identified, Tukey's honestly significant difference test was applied for post-hoc multiple comparisons. Categorical variables were analyzed using the χ^2^ test. To determine independent predictors of systemic arterial stiffness, measured by CAVI, both univariate and multivariate linear regression analyses were performed. The initial full multivariable model incorporated clinically relevant covariates, including age, sex, SBP, and DBP, along with all primary LSFG parameters (BS, BOM, and LF) for both nasal and temporal regions. These covariates were selected a priori because age, sex, and blood pressure are established determinants of systemic arterial stiffness, whereas BS, BOM, and LF were included to assess whether pulsatility-, resistance-, and autonomic-related ocular hemodynamic parameters provide additional explanatory value for CAVI. The initial full multivariable model was considered the primary inferential model. Subsequently, a stepwise variable selection method, based on minimization of the Akaike information criterion, was used secondarily as an exploratory approach to derive a parsimonious candidate model. Multicollinearity diagnostics were performed for the variables retained in the final selected model using variance inflation factors (VIFs). A two-tailed *P*-value < 0.05 was considered statistically significant.

## Results

### Baseline Characteristics of the Study Cohort

A total of 72 patients were included in the study. The baseline demographic, ocular, and systemic characteristics of the cohort, stratified by DR severity, are presented in Table 1. Statistically significant differences were identified among the DR severity groups concerning age (*P* < 0.01) and BMI (*P* = 0.02); however, no significant difference was noted in sex distribution (*P* = 0.72). Notably, post-hoc analysis using Tukey's honestly significant difference test revealed that the mild NPDR group was significantly older (mean age: 77.4 ± 7.0 years) than the PDR group (mean age: 57.3 ± 12.5 years, *P* < 0.01). Although the overall analysis of variance indicated a significant difference in BMI, post-hoc pairwise comparisons did not yield statistical significance between any specific pairs of groups. Although CAVI values exhibited variability across groups, no statistically significant difference was found among the DR severity stages (*P* = 0.08; Table 1, Fig. 2). Other systemic blood pressure and laboratory findings were comparable across groups.

**Table 1.** Baseline Characteristics of the 72 Studied Patients

	Whole (*N* = 72)	NDR (*N* = 10)	Mild NPDR (*N* = 9)	Moderate NPDR (*N* = 9)	Severe NPDR (*N* = 5)	PDR (*N* = 39)	*P*
Sex							0.72
Male	41	5	5	4	2	25	
Female	31	5	4	5	3	14	
Age (years)	61.2 ± 14.3	61.9 ± 21.3	77.4 ± 7.0	63.8 ± 9.8	57.4 ± 8.6	57.3 ± 12.5^*^	<0.01^†^
LogMAR VA	0.3 ± 0.4	0.1 ± 0.2	0.2 ± 0.2	0.1 ± 0.1	0.2 ± 0.3	0.4 ± 0.5	0.15
IOP (mm Hg)	15.8 ± 3.7	17.4 ± 4.5	12.7 ± 1.2	15.3 ± 4.4	17.0 ± 3.1	16.1 ± 3.5	0.05
SBP (mm Hg)	146 ± 21.8	145± 32.9	153 ± 16.7	143± 18.4	141 ± 9.5	146± 22.8	0.90
DBP (mm Hg)	79.1 ± 14.2	75.6 ± 13.0	70.9 ± 5.2	79.1 ± 11.8	85.4 ± 10.5	80.7 ± 16.1	0.34
MBP (mm Hg)	101 ± 13.8	95.8 ± 14.7	98.1 ± 6.1	100 ± 11.0	105 ± 11.1	102 ± 15.7	0.76
OPP (mm Hg)	51.8 ± 9.3	46.7 ± 7.3	52.9 ± 4.7	52.0 ± 8.9	53.1 ± 5.3	52.2 ± 10.6	0.73
BMI	25.4 ± 4.5	29.6 ± 7.2	24.5 ± 2.6	25.8 ± 3.1	30.7 ± 1.6	24.5 ± 4.3	0.02^†^
CAVI (Right)	9.4 ± 1.5	9.0 ± 1.2	10.1 ± 2.5	10.4 ± 1.4	8.4 ± 0.7	9.2 ± 1.2	0.08
HbA1c (%)	7.8 ± 1.7	8.2 ± 2.7	8.0 ± 1.4	7.2 ± 1.1	8.2 ± 1.9	7.8 ± 1.7	0.74
LDL (mg/dL)	111 ± 41.1	107 ± 39.2	110 ± 26.9	99.0 ± 18.8	119 ± 29.3	114 ± 49.0	0.91
HDL (mg/dL)	56.5 ± 20.4	65.2 ± 33.3	53.2 ± 11.3	51.9 ± 17.3	58.3 ± 27.8	56.1 ± 19.0	0.73
BUN (mg/dL)	19.3 ± 10.2	15.0 ± 4.7	19.3 ± 4.9	21.1 ± 13.6	14.1 ± 3.9	20.4 ± 11.5	0.55
Cre (mg/dL)	1.2 ± 1.6	1.3 ± 1.6	0.8 ± 0.4	0.8 ± 0.5	0.6 ± 0.1	1.5 ± 1.9	0.65

BUN, blood urea nitrogen; Cre, creatinine; HbA1c, glycated hemoglobin; HDL, high-density lipoprotein cholesterol; IOP, intraocular pressure; LDL, low-density lipoprotein cholesterol; LogMAR, logarithm of the minimum angle of resolution; MBP, mean blood pressure; NDR, no diabetic retinopathy; OPP, ocular perfusion pressure; VA, visual acuity.

* *P* < 0.01 vs. mild NPDR group by Tukey's honestly significant difference test.

† One-way analysis of variance.

**Figure 2.**
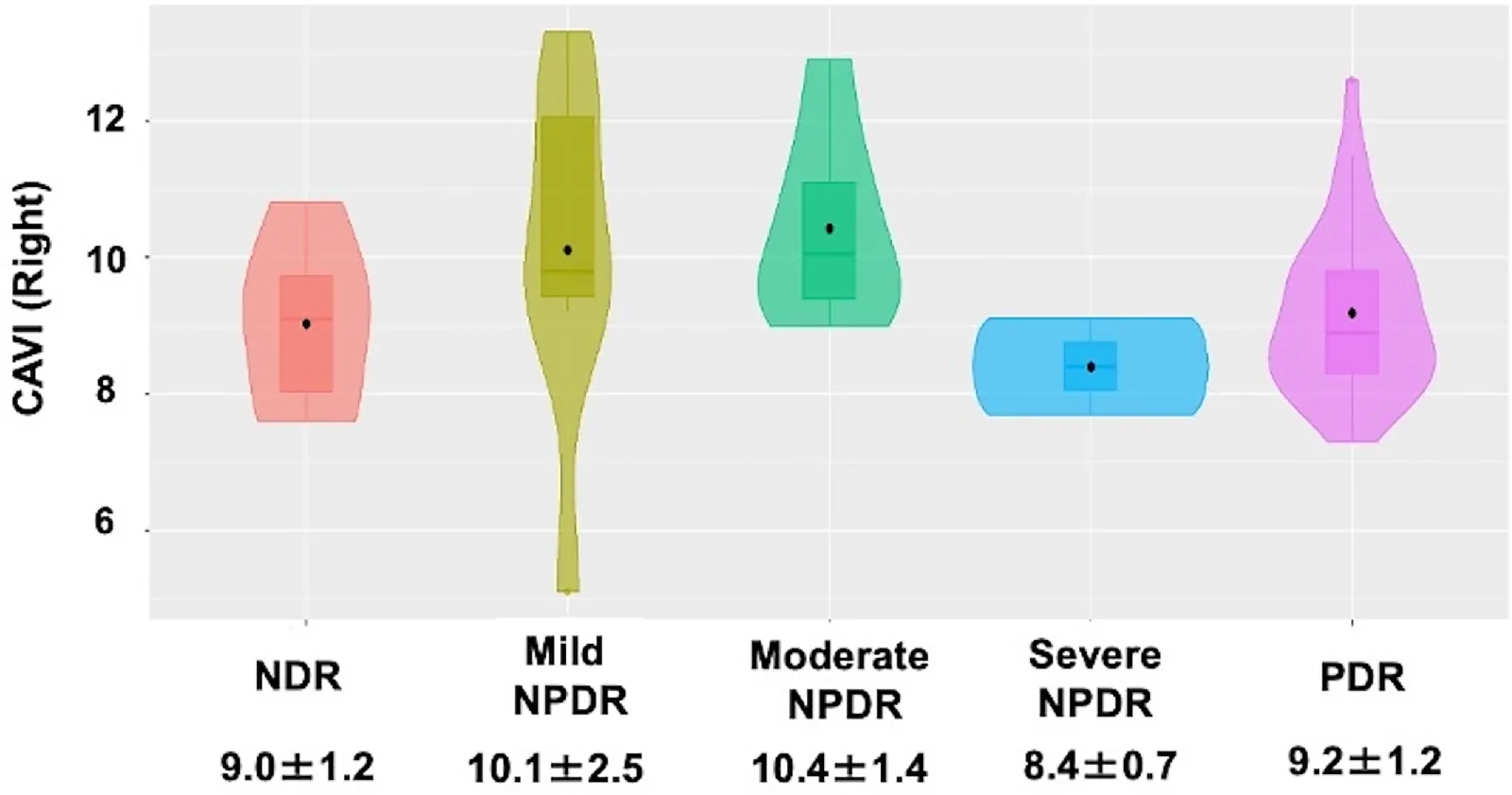
Violin plots depicting the distribution of CAVI values across the five DR severity groups: NDR (*n* = 10), mild NPDR (*n* = 9), moderate NPDR (*n* = 9), severe NPDR (*n* = 5), and PDR (*n* = 39). The *dots* represent the median values, and the *boxes* indicate the interquartile ranges. Although the CAVI values varied, no statistically significant difference was observed among the groups (*P* = 0.08, one-way analysis of variance). NDR, no diabetic retinopathy.

### Univariate and Multivariable Predictors of CAVI

Univariate linear regression analysis identified several factors significantly associated with CAVI (Table 2). A significant positive association was observed between higher CAVI and older age (estimate = 0.06, *P* < 0.01). Among the choroidal hemodynamic parameters, significant positive associations with CAVI were noted for nasal pulsatility (BS_N; *P* = 0.01), nasal resistance (BOM_N; *P* = 0.03), temporal pulsatility (BS_T; *P* < 0.01), and temporal resistance (BOM_T; *P* < 0.01). Conversely, systemic blood pressure parameters (SBP and DBP) showed no significant associations with CAVI.

**Table 2.** Univariable Linear Regression Analysis With CAVI as the Dependent Variable

Parameters	Estimate	*P*	Conf.Low	Conf.High
Sex	0.462	0.34	−0.499	1.424
Age	0.06	<0.01	0.033	0.087
BS_N	0.16	0.01	0.04	0.281
BS_T	0.199	<0.01	0.098	0.3
BOM_N	1.431	0.03	0.173	2.689
BOM_T	2.383	<0.01	1.114	3.652
LF_N	0.215	0.41	−0.301	0.732
LF_T	0.215	0.26	−0.167	0.597
SBP	0	0.99	−0.025	0.024
DBP	−0.025	0.14	−0.058	0.008

LF, low-frequency component of the mean blur rate waveform; N, nasal peripapillary region; T, temporal peripapillary region.

In the final exploratory stepwise multiple regression model, age remained significantly associated with higher CAVI (β = 0.048, *P* = 0.020) (Table 3). Notably, two regional choroidal vascular resistance parameters were retained in the final model: temporal BOM (BOM_T) demonstrated a positive trend with CAVI (β = 2.719, *P* = 0.055), whereas nasal BOM (BOM_N) exhibited a negative trend (β = −2.054, *P* = 0.070). The final model was highly significant, explaining 32.6% of the variance in CAVI (Adjusted *R*² = 0.326, *P* < 0.001). Multicollinearity diagnostics for the final selected model showed VIF values of 2.32, 4.26, and 5.53, indicating no marked multicollinearity, although one variable slightly exceeded the conventional threshold of 5.

**Table 3.** Multiple Regression Analysis of Factors Associated With CAVI

			Final Stepwise Model		
Variable	Initial Full Model β (95% CI)	*P* value	β	*P* Value	VIF
Age (years)	0.035 (−0.017 to 0.086)	0.18	0.048	0.020	2.32
Sex (male)	0.515 (−0.421 to 1.45)	0.272	—	—	
SBP (mm Hg)	−0.005 (−0.032 to 0.021)	0.683	—	—	
DBP (mm Hg)	0.014 (−0.028 to 0.055)	0.508	—	—	
BS_N	−0.033 (−0.272 to 0.206)	0.782	—	—	
BS_T	0.100 (−0.055 to 0.255)	0.197	—	—	
BOM_N	−1.84 (−4.69 to 1.01)	0.2	−2.054	0.070	4.26
BOM_T	2.86 (−0.688 to 6.42)	0.111	2.719	0.055	5.53
LF_N	−0.236 (−0.882 to 0.411)	0.465	–	–	
LF_T	0.052 (−0.463 to 0.568)	0.838	–	–	

CI, confidence interval.

## Discussion

In this retrospective cross-sectional study of patients with type 2 diabetes across DR stages, age was the strongest determinant of CAVI. Regional choroidal BOM parameters were retained only in the exploratory stepwise model, suggesting a possible ocular–systemic vascular relationship. The strengths of our study include the use of BOM as a novel LSFG-derived resistance-related waveform index, direct comparison with a validated marker of arterial stiffness (CAVI), and a region-specific analysis of the peripapillary choroid.

The biological link between systemic arterial stiffness and choroidal vascular resistance is well established. Previous studies have shown that a higher CAVI correlates with a lower choroidal vascularity index, indicating structural vascular dropout in patients with diabetes.^10^ Our findings complement this evidence by demonstrating functional alterations, evidenced by increased vascular resistance. Stiff central arteries lose their Windkessel buffering function, which results in excessive pulsatile load being transmitted to the ocular microvasculature. Because of its high-flow characteristics and relatively weak autoregulatory capacity compared with the retinal circulation, the choroid is particularly susceptible to such mechanical stress. This vulnerability may exacerbate pre-existing diabetic microvascular damage, including basement membrane thickening and choriocapillaris degeneration.^28^^,^^29^

Notably, temporal choroidal vascular resistance was retained as an explanatory variable in the final exploratory stepwise model. The temporal–nasal difference should be interpreted cautiously and may reflect regional heterogeneity in peripapillary choroidal circulation. The macular region contains several choroidal watershed zones that may be more susceptible to lower perfusion pressure and flow instability.^26^ Our recent study using LSFG further showed that the spatial pattern of the choroidal hypoperfusion zone varied among eyes, with a predominantly temporal distribution being more common than a symmetric distribution around the ONH.^25^ Taken together, these findings suggest that the present temporal–nasal difference may, at least in part, reflect variation in the spatial relationship between the ONH and the surrounding watershed-related hypoperfusion zone.

Consistent with some previous reports, we did not observe a significant linear association between CAVI and DR severity. Previous studies have reported associations between systemic arterial stiffness or atherosclerosis and DR, although results may vary according to the population and study design.^11^^,^^30^ In our cohort, there were substantial imbalances in baseline characteristics—particularly age and BMI—across the DR severity groups. Both factors are known to independently influence CAVI: it increases with age^31^ and demonstrates an inverse relationship with BMI.^32^ Notably, the PDR group in our study was significantly younger than the mild NPDR group, a pattern that likely masked any potential relationship between DR severity and arterial stiffness. These confounding effects highlight the necessity of adjusting for demographic and metabolic variables and suggest that while arterial stiffness is a well-established predictor of cardiovascular events,^33^ it may not serve as a direct, linear indicator of DR stage.

This study has several limitations. First, its retrospective, cross-sectional design precludes causal inference. Second, the absence of age-matching across DR severity groups introduces confounding; however, we addressed this issue through multivariable adjustment. Third, as this is an exploratory analysis, no formal sample size calculation was conducted. In addition, subgroup sizes were unbalanced across DR severity stages, particularly for severe NPDR, which may have reduced statistical power for stage-based comparisons and contributed to imprecision in regression estimates. Larger, more balanced cohorts will be crucial for validating the observed BOM trends. Fourth, unmeasured systemic factors, including medication profiles, may influence vascular stiffness. Fifth, because this was a cross-sectional study using one prespecified eye per participant, longitudinal baseline-based normalization and within-eye change assessment were not feasible, and we therefore prioritized interpretation based on physiologically defined parameters and effect estimates rather than additional post hoc normalized metrics. Sixth, ROI placement was performed manually by a single retina specialist using a predefined template-based protocol; however, some degree of operator-dependent subjectivity in ROI selection cannot be excluded. Seventh, BOM is an indirect LSFG-derived resistance-related index rather than a direct physical measurement of vascular resistance; therefore, its physiological interpretation remains inferential. Finally, LSFG and CAVI were not measured simultaneously. Although we restricted paired measurements to a three-month window to improve clinical comparability, temporal variability in systemic and ocular hemodynamics may have diminished the observed associations. Prospective studies with same-day ocular and systemic measurements will be necessary for definitive validation. In addition, selection bias and limited generalizability are also potential concerns because of the single-center design.

In conclusion, age was the most robust determinant of CAVI in this cohort. The retention of regional choroidal BOM parameters in the exploratory stepwise model suggests a possible ocular–systemic vascular relationship, whereby systemic pulsatile stress may influence choroidal hemodynamics; however, these findings are hypothesis-generating. Larger prospective studies with same-day ocular and systemic measurements are needed to validate these findings and clarify their clinical relevance.
